# Identification of reference genes for RT-qPCR data normalisation in aging studies

**DOI:** 10.1038/s41598-019-50035-0

**Published:** 2019-09-27

**Authors:** Lourdes González-Bermúdez, Teresa Anglada, Anna Genescà, Marta Martín, Mariona Terradas

**Affiliations:** 1 Departament de Biologia Cel·lular, Fisiologia i Immunologia, Facultat de Biociències, Universitat Autònoma de Barcelona, Bellaterra, Spain; 2 Present Address: Hereditary Cancer Program, Catalan Institute of Oncology, IDIBELL, Hospitalet de Llobregat, Barcelona, Spain

**Keywords:** Senescence, Ageing

## Abstract

Aging is associated with changes in gene expression levels that affect cellular functions and predispose to age-related diseases. The use of candidate genes whose expression remains stable during aging is required to correctly address the age-associated variations in expression levels. Reverse transcription quantitative-polymerase chain reaction (RT-qPCR) has become a powerful approach for sensitive gene expression analysis. Reliable RT-qPCR assays rely on the normalisation of the results to stable reference genes. Taken these data together, here we evaluated the expression stability of eight frequently used reference genes in three aging models: oncogene-induced senescence (OIS), *in vitro* and *in vivo* aging. Using NormFinder and geNorm algorithms, we identified that the most stable reference gene pairs were *PUM1* and *TBP* in OIS, *GUSB* and *PUM1* for *in vitro* aging and *GUSB* and *OAZ1* for *in vivo* aging. To validate these candidates, we used them to normalise the expression data of *CDKN1A*, *APOD* and *TFRC* genes, whose expression is known to be affected during OIS, *in vitro* and *in vivo* aging. This study demonstrates that accurate normalisation of RT-qPCR data is crucial in aging research and provides a specific subset of stable reference genes for future aging studies.

## Introduction

Aging is a complex physiological process that affects organismal, tissue and cellular levels, and it is characterised by a persistent loss of cellular and tissue integrity that leads to impaired biological function and increased risk of pathologies and diseases^1^. Hayflick and Moorehead^2^ proved that normal cells have limited proliferation capability in culture and enter senescence due to the loss of telomeric integrity, after extensive replicative shortening, or the accumulation of unrepairable DNA damage^3,4^. Since then, studies using cells aged *in vitro* after extensive passaging have been widely used in aging research. Additionally, fibroblast strains, such as BJ or IMR90, have been used to generate stable models of inducible senescence in which the proliferative arrest involves the activation of both the retinoblastoma and p53 pathways, by an activating mutation of an oncogene, termed as oncogene induced senescence (OIS)^5^. Living organisms also produce senescent cells in their tissues, whose frequency increases with age^1^ and contributes to the age-associated decline of regenerative capacity, tissue inflammation and the development of age-related diseases^6,7^. While *in vitro* induced senescent cells are almost homogeneous populations, in  *in vivo* aged tissues, senescent cells coexist with young cells and with old cells that have accumulated many divisions but have not yet reached senescence. Thus, cells derived from old individuals share cellular and molecular phenotypes with *in vitro* senescent cells, but it is not clear whether these phenotypes are completely overlapping^8^. For this reason, when possible, researchers use *in vivo* model studies in which cells from young and elderly donors are used immediately after obtention or after a few passages in culture. Although these are the currently used models or approaches to study aging, careful consideration must be given to the differences among them.

Profound changes in gene expression are important determinants of organismal aging^9,10^, and many genes change their expression with age^8,11–13^. Therefore, understanding gene expression changes provides insights into the molecular mechanisms underlying normal and pathological aging processes. Genome-wide studies in different species have identified sets of genes displaying age-associated expression alterations as promising biomarkers of aging^4,14^. For instance, a meta-analysis of the age-associated gene expression profile has identified genes involved in the stress response, such as hypoxia-inducible factor 1-alpha (*HIF1A*) or apolipoprotein D (*APOD)*, and in cell cycle regulation and apoptosis, such as cyclin dependent kinase inhibitor 1A (*CDKN1A*) and annexins, to be upregulated in a wide variety of tissues from humans, mice and rats during *in vivo* aging^12^. Other *in vivo* aging studies in mammals have identified the downregulation of genes mainly associated with metabolism and mitochondrial function^12,15,16^.

In contrast, age-associated variations in gene expression may also reflect the selected aging model. For example, it is possible to distinguish the molecular phenotype of human fibroblasts that have acquired the senescent phenotype *in vitro* from that of fibroblasts obtained from old individuals^8^. A cluster analysis from a cDNA microarray that evaluated the expression of 384 cancer-related genes in three types of aging: (i) replicative senescent fibroblasts, (ii) fibroblasts from a progeria patient and (iii) primary fibroblasts from an elderly donor, revealed that replicative senescent cells were clearly different from the other two aging models^17^. In a correlation-based comparison of gene expression in microarray datasets from senescent and *in vivo* aged human and mouse cells, similar expression signatures between the cellular senescence” and *in vivo* aged cells could be established in mouse, but not in human cells. In conclusion, gene expression varies with age, but this variation is also influenced by the aging model used in the study^18^.

A powerful tool used to evaluate changes in gene expression is the reverse transcription quantitative-polymerase chain reaction (RT-qPCR) technique. Features including great accuracy, high sensitivity, reproducibility and high-throughput make RT-qPCR the most prevalent technique to assess mRNA expression^19,20^. Relative quantification of data obtained from RT-qPCR is used to determine changes in gene expression across multiple samples or conditions after normalisation to an internal reference gene^20^. Thus, the accuracy of RT-qPCR relies on the availability of reference genes that maintain stable expression levels under the tested conditions, which could be used as endogenous controls for normalisation^21,22^. Consequently, identification of appropriate reference genes is a crucial step in the correct development of RT-qPCR assays.

The ideal reference gene should exhibit stable expression in different cell types, tissues and experimental conditions or treatments^21^. Traditionally, genes related to basal cellular function have been extensively used as reference genes^23^. Nevertheless, numerous studies have demonstrated that the expression of reference genes varies under different conditions, reflecting the importance of validating the expression stability of the selected reference genes under the desired experimental settings before performing the RT-qPCR assays^24–26^. Focusing on human aging, frequently used reference genes such as β-2 microglobulin (*β2M*) showed age-dependent variation with increased expression in aged muscle cells^27^. In addition, glucuronidase beta (*GUSB*) was found to be the most suitable reference gene, whereas *18s* *rRNA* was found to be the least stable gene for expression studies in peripheral blood mononuclear cells from young and aged subjects^28^. Recently, transmembrane protein 199 (*TMEM199*) has been described as a new candidate reference gene to normalise RT-qPCR data of senescent cells^29^. These findings reveal differences in gene expression associated with species, tissues and age models and bring to light the difficulties in finding a stable reference gene for expression studies during aging. Therefore, the purpose of this study is to identify a set of stable reference genes that are suitable for the analysis of human age-associated gene expression changes in different models of aging.

To accomplish this goal, we analysed the impact of OIS, *in vitro* and *in vivo* aging on the expression stability of eight commonly used reference genes that play a role in different biological functions and that have been previously tested in age-related studies^23,27–30^: glyceraldehyde-3-phosphate dehydrogenase (*GAPDH*) and *GUSB* are involved in basal cellular functions; β-actin (*ACTB*) is an essential component of the cytoskeleton^31^; hypoxanthine guanine phosphoribosyl transferase (*HPRT1*) participates in nucleotide synthesis^32^; pumilio-homolog 1 (*PUM1*) is involved in translation regulation^33^; TATA box binding protein (*TBP*) functions in transcription regulation^34^; ornithine decarboxylase antizyme 1 (*OAZ1*) is related to protein biosynthesis^35^; *TMEM199* is involved in Golgi homeostasis^36^. To analyse the expression stability of the candidate reference genes and to select the genes that are most suitable, we used the statistical software packages NormFinder^37^ and geNorm^38^. Finally, to validate their reliability, we evaluated the expression of genes that are known to change with aging after normalisation with the selected candidate reference genes.

## Results

### Establishment of senescence and aging models and testing of primers for reference genes

To determine the impact of aging on the stability of reference genes, we employed three aging models. The first model examined was the OIS model, which was established in immortalised primary human BJ foreskin fibroblasts (BJ fibroblasts) transduced with the *H-RAS* oncogene fused to a 4-hydroxytamoxifen (4-OHT)-responsive Estrogen Receptor (ER) ligand binding domain^39^. Upon eight days of 4-OHT treatment, the cells were stained for senescence-associated beta-galactosidase (SA-βgal) activity. The percentage of BJ fibroblasts displaying SA-βgal activity after 4-OHT treatment was significantly higher (60.25%) compared to the control conditions (2.85%) (Chi-square test; *p* < 0.001) (Fig. S1). The second model employed was the *in vitro* aging model, which consists of the serial culture of primary human dermal fibroblasts (HDFs), allowing the analysis of gene expression in a constant environment over time^40^. Primary HDFs have a limited growth potential and reach their replicative limit after 30 passages in culture^41^. Thus, we established a distinction between young cells (early passage, EP), which were cells with less than 10 passages in culture, and old cells (late passage, LP), which were cells with more than 20 passages in culture. Finally, we used Human Mammary Epithelial Cells (HMECs) derived from the healthy tissue of young (≤19 years of age) and old (≥61 years of age) donors as a model for *in vivo* aging.

**Figure 1:**
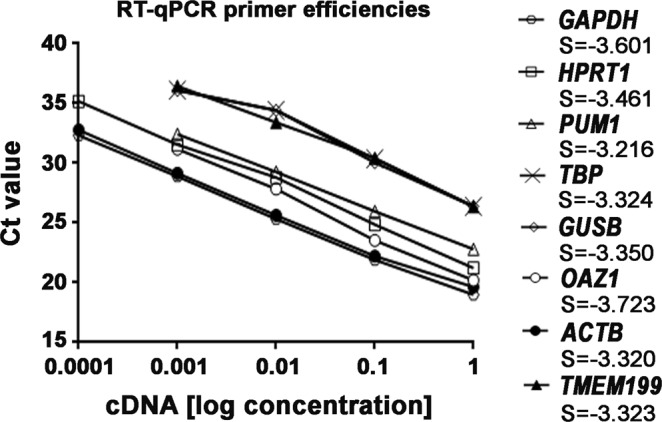
Evaluation of RT-qPCR primer efficiencies. The amplification efficiency for each primer threshold cycle (Ct) and the logarithm of the initial cDNA concentrations were plotted to calculate the slope (S) of each primer pair. Standard curves were generated from at least four dilution points for each primer pair. RT-qPCR reactions for each sample were run in duplicate, with standard deviations <0.85.

To assess the RT-qPCR assay performance, we first calculated the amplification efficiency for all the primer pairs analysed in this study. RT-qPCR primer efficiencies were obtained from the slopes of their corresponding standard curves (Fig. 1, Table 1). Primer efficiencies ranged from 85.61% to 104.61%, values included in the optimal efficiency range^42,43^, and standard curves showed high linearity with correlation coefficients (R^2^) between 0.971 and 0.999 (Table 1). Evaluation of the melting curves revealed single peaks for each primer pair, which confirmed the specificity of the amplifications (Fig. S2).

**Table 1:** List of the reference genes tested by RT-qPCR.

Gene Symbol	Accession Number	Primer Sequence	Amplicon Size (bp)	Efficiency (%)	R^2^
*GAPDH*	NM_002046	F:AGCCACATCGCTCAGACAC R:GCCCAATACGACCAAATCC	66	98.43	0.998
*HPRT1*	NM_000194	F:GGCCAGACTTTGTTGGATTTG R:TGCGCTCATCTTAGGCTTTGT	144	104.61	0.999
*PUM1*	NM_001020658	F:CGGTCGTCCTGAGGATAAAA R: CGTACGTGAGGCGTGAGTAA	121	94.48	0.997
*TBP*	NM_003194	F:GAGAGTTCTGGGATTGTACCG R:ATCCTCATGATTACCGCAGC	143	99.89	0.971
*GUSB*	NM_000181	F:AAGTCCTTCACCAGCAGCG R:CCACGGTGTCAACAAGCAT	75	98.84	0.972
*OAZ1*	NM_004152	F:GGATCCTCAATAGCCACTGC R:TACAGCAGTGGAGGGAGACC	150	85.61	0.997
*ACTB*	NM_001101	F: CCAACCGCGAGAAGATGA R: CCAGAGGCGTACAGGGATAG	97	100.05	0.996
*TMEM199*	NM_152464	F: CACCAGCATCTGAGAGAAAGG R: CCGTGGAGGCTTCACAAC	96	99.95	0.994

Abbreviations: bp, base pair; F, forward; R, reverse; R^2^, correlation coefficient of the corresponding standard curve.

### Descriptive statistics of candidate reference genes

To study the expression profile of the candidate reference genes in the different aging models, RT-qPCR experiments were performed and the mean cycle threshold (Ct) value for each gene was calculated and the variation in expression among different samples, defined as the coefficient of variation (CV), was obtained for each gene. The CV should be small for a reference gene; thus, we considered that a stable and reliable reference gene should have a CV lower than 2.

**Figure 2:**
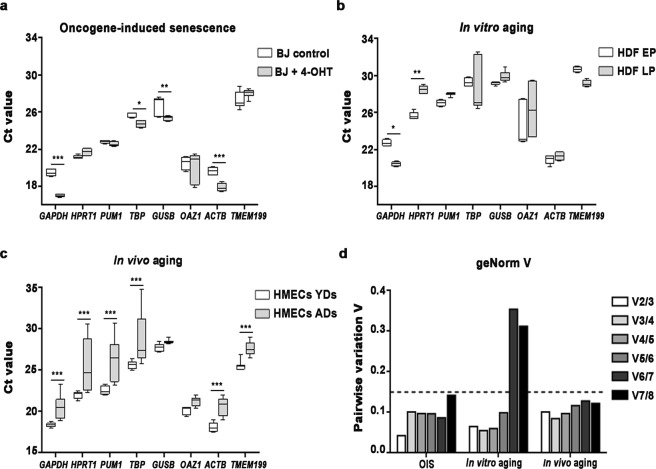
Ct values of eight candidate reference genes in different aging models and the optimal number of reference genes needed for accurate normalisation calculated by geNorm. Box and whisker plots show the raw Ct values of the candidate reference genes during (**a**) oncogene-induced senescence in BJ fibroblasts, (**b**) *in vitro* aging in HDFs and (**c**) *in vivo* aging in HMECs from young and aged donors. (**d**) Pairwise variation (V_n/n+1_) of candidate reference genes was obtained by geNorm to determine the required number of reference genes for accurate normalisation in each aging model. A discontinuous line indicates a pairwise variation (V) of 0.15, the cut-off value defined by geNorm. Abbreviations: 4-OHT: 4-Hydroxytamoxifen; Ct: cycle threshold; HDFs: Human Dermal Fibroblasts; HMECs: Human Mammary Epithelial Cells; EP: early passage (<10); LP: late passage (>20); YDs: young donors; ADs: aged donors. Notes: the boxes include values from the 25th to the 75th percentiles, the line across the box indicates the median, and whiskers show the minimum and maximum values for each reference gene. One-way ANOVA test was conducted. In those cases were *p-*values were < 0.05, a Bonferroni post-hoc test was conducted. Bonferroni corrected *p*-values are shown (*p < 0.05, **p < 0.01, ***p < 0.001).

In the OIS model, most reference genes showed very few intragroup expression variations, with *PUM1*, *HPRT1* and *TBP* being the reference genes that exhibited the most stable expression levels (CV < 2 for control and senescent BJ fibroblasts), as Ct values remained constant within control and senescent cells (Fig. 2a). *OAZ1* and *TMEM199* displayed strong intragroup variations, showing the highest CV values (Fig. 2a). In senescent BJ fibroblasts, *GAPDH* and *ACTB* showed the strongest significant decrease in their expression level in comparison to control fibroblasts. To compare variations in reference genes expression with senescence, a one-way between subjects ANOVA using the Ct values of each reference gene was conducted, revealing a significant effect of OIS on reference gene expression [F (15, 76) = 2.045, *p* = 0.0224]. *GAPDH* expression levels varied between control (mean Ct values: 19.48; standard deviation (SD: 0.40) and senescent BJ fibroblasts (mean Ct values: 17.11; SD: 0.21) (Bonferroni post-hoc test; *p* < 0.001) (Fig. 2a). Similarly, expression levels of *ACTB* displayed low intragroup variations, but they significantly differed between control (mean Ct value: 19.66; SD: 0.42) and senescent BJ fibroblasts (mean Ct value: 17.90; SD: 0.44) (Bonferroni post-hoc test; *p* < 0.001) (Fig. 2a). These results point to *PUM1*, *HPRT1* and *TBP* as the most stable reference genes for expression studies during OIS.

Regarding the expression of candidate reference genes in *in vitro* aged HDFs, we found *GUSB* and *PUM1* to have the lowest intragroup expression variations, reflected by the smallest CV values in EP and LP HDFs (Fig. 2b). Conversely, the highest variable intragroup expression levels were detected for *OAZ1* and *TBP* (CV > 2) (Fig. 2b). We also detected a significant effect of the *in vitro* age of HDFs on reference gene expression after conducting a one-way ANOVA test [F (15, 73) = 2.337, *p* = 0.0086]. The Bonferroni post-hoc test showed that the expression level of *GAPDH* significantly varied between early (mean Ct value: 22.74; SD: 0.33) and late (mean Ct value: 20.46; SD: 0.23) passage HDFs (*p* = 0.0177) (Fig. 2b). Expression level of *HPRT1* was also found to significantly vary between EP (mean Ct value: 25.65; SD: 0.41) and LP (mean Ct value: 28.48; SD: 0.42) HDFs (Bonferroni post-hoc test; *p* = 0.0048) (Fig. 2b). Therefore, *GUSB* and *PUM1* seem to be the most suitable reference genes for expression analysis in *in vitro* aging models.

The expression profile of the candidate reference genes suffered from strong intragroup variations in HMECs from older donors, as almost all the reference genes presented a large intragroup Ct variability in aged donors (CV > 2) (Fig. 2c). However, the strongest intra- and intergroup variations were observed for *TBP*. A significant effect of the donor’s age of HMECs on reference gene expression was also found after applying a one-way ANOVA test [F (15, 239) = 9.936, *p* < 0.001]. The post-hoc comparisons using the Bonferroni test indicated that the mean Ct value significantly increased in HMECs from aged donors (mean Ct value: 25.65; SD: 0.47) when compared to HMECs from young women (mean Ct value: 28.81; SD: 2.962) (one-way ANOVA and Bonferroni multiple correction test; *p* < 0.001). Furthermore, the mean Ct values of *GAPDH*, *HPRT1*, *PUM1*, *ACTB* and *TMEM199* were also found to increase significantly in HMECs from aged donors in comparison to HMECs from younger donors (Bonferroni post-hoc test; *p* < 0.001) (Fig. 2c). Only *GUSB* and *OAZ1* expression levels showed very little intragroup variation, with no significant differences between HMECs from young and aged donors (Fig. 2c). Therefore, these two genes appeared to be the most stable reference genes to study *in vivo* aging.

### Expression stability of candidate reference genes

To select appropriate reference genes for each different aging model, genes were ranked based on their expression stability, which was evaluated with two statistical algorithms, NormFinder^37^ and geNorm^38^. It should be noted that the ranking of reference genes showed similar trends between both software packages, although with subtle variations, which might be attributed to the differences across algorithms. However, when faced with discrepancies between these methods, the recommended genes are always those closest to the most stable genes, reflecting their ability to be a suitable reference gene.

In OIS, both NormFinder and geNorm revealed that the least stable gene was *OAZ1*, followed by *TMEM199* (Table 2). These genes displayed high levels of intragroup variation in their expression profile (Fig. 2a). On the contrary, NormFinder and geNorm indicated that the most suitable genes to use as a reference gene were *PUM1*, followed by *TBP* and *HPRT1* (Table 2). This classification is in accordance with the expression profiles that we previously obtained (Fig. 2a) in which these reference genes were those with the lowest intragroup variability and their expression levels were very similar before and during senescence.

**Table 2:** Candidate reference gene expression stability ranked by NormFinder and geNorm in different aging processes. Putative reference genes are listed from top to bottom in order of decreasing stability for each aging process.

Aging Process	Rank	NormFinder		geNorm	
		Gene	Stability value	Gene	M
OIS	1	*PUM1*	0.097	*PUM1*	0.507
	2	*TBP*	0.109	*HPRT1*	0.553
	3	*GUSB*	0.111	*TBP*	0.570
	4	*GAPDH*	0.112	*GAPDH*	0.592
	5	*HPRT1*	0.144	*GUSB*	0.598
	6	*ACTB*	0.204	*ACTB*	0.673
	7	*TMEM199*	0.208	*TMEM199*	0.732
	8	*OAZ1*	0.343	*OAZ1*	1.179
*In vitro* aging	1	*PUM1*	0.024	*PUM1*	0.945
	2	*GUSB*	0.039	*GUSB*	0.948
	3	*GAPDH*	0.040	*GAPDH*	0.951
	4	*TMEM199*	0.095	*TMEM199*	0.964
	5	*HPRT1*	0.148	*HPRT1*	1.061
	6	*TBP*	0.251	*ACTB*	1.283
	7	*ACTB*	0.401	*TBP*	2.288
	8	*OAZ1*	0.408	*OAZ1*	2.582
*In vivo* aging	1	*OAZ1*	0.104	*GUSB*	0.565
	2	*GUSB*	0.105	*ACTB*	0.574
	3	*ACTB*	0.110	*OAZ1*	0.583
	4	*HPRT1*	0.111	*PUM1*	0.630
	5	*TMEM199*	0.116	*GAPDH*	0.716
	6	*PUM1*	0.123	*TMEM199*	0.874
	7	*TBP*	0.127	*HPRT1*	0.994
	8	*GAPDH*	0.145	*TBP*	1.077

Abbreviations: OIS, oncogene-induced senescence; M, expression stability value.

The evaluation of gene stability during *in vitro* aging after applying NormFinder and geNorm agreed in suggesting that *OAZ1* was the least stable gene, together with *TBP* and *ACTB* (Table 2). In accordance to their expression profiles, these genes were those with higher levels of intragroup variation (Fig. 2b). Additionally, analysis with NormFinder and geNorm rendered *PUM1* > *GUSB* > *GAPDH* as the most stable genes (Table 2). In agreement with the above results, expression profiles of *GUSB* and *PUM1* were very similar in young and in *in vitro*-aged fibroblasts (Fig. 2b). Instead, *GAPDH* levels were very stable within each group, although they significantly decreased during *in vitro* aging, a probable reason for its third position in the ranking (Fig. 2b).

For *in vivo* aging studies, NormFinder ranked *GAPDH* < *TBP* < *PUM1* as the least stable genes, while geNorm designated *TBP* < *HPRT1* < *TMEM199* (Table 2). Regarding the most stable genes, NormFinder identified *OAZ1* > *GUSB* > *ACTB*, whereas geNorm showed *GUSB* > *ACTB* > *OAZ1* (Table 2). Consistent with our earlier results, the expression profiles of *OAZ1* and *GUSB* showed stable intragroup levels and no expression differences between HMECs from young and aged donors (Fig. 2c).

Finally, an optimal number of reference genes (n) for accurate normalisation was calculated by comparing the pairwise variation (V) of two sequential normalisation factors (V_n_/_n+1_) with geNorm. In all aging processes, V_2/3_ < 0.15 indicated that two reference genes, among those selected by the statistical algorithms, were enough to accurately normalise RT-qPCR data (Fig. 2d).

### Validation of selected reference genes

To validate the reference genes selected by the statistical algorithms as candidates for normalisation, we selected genes whose expression levels have been shown to change with senescence or aging. For each aging process, data were normalised to three distinct groups of reference genes. In agreement with the outcomes of NormFinder and geNorm algorithms, we selected the single most stable gene, the recommended combination of two reference genes and the candidate reference gene with the lowest stability.

**Figure 3:**
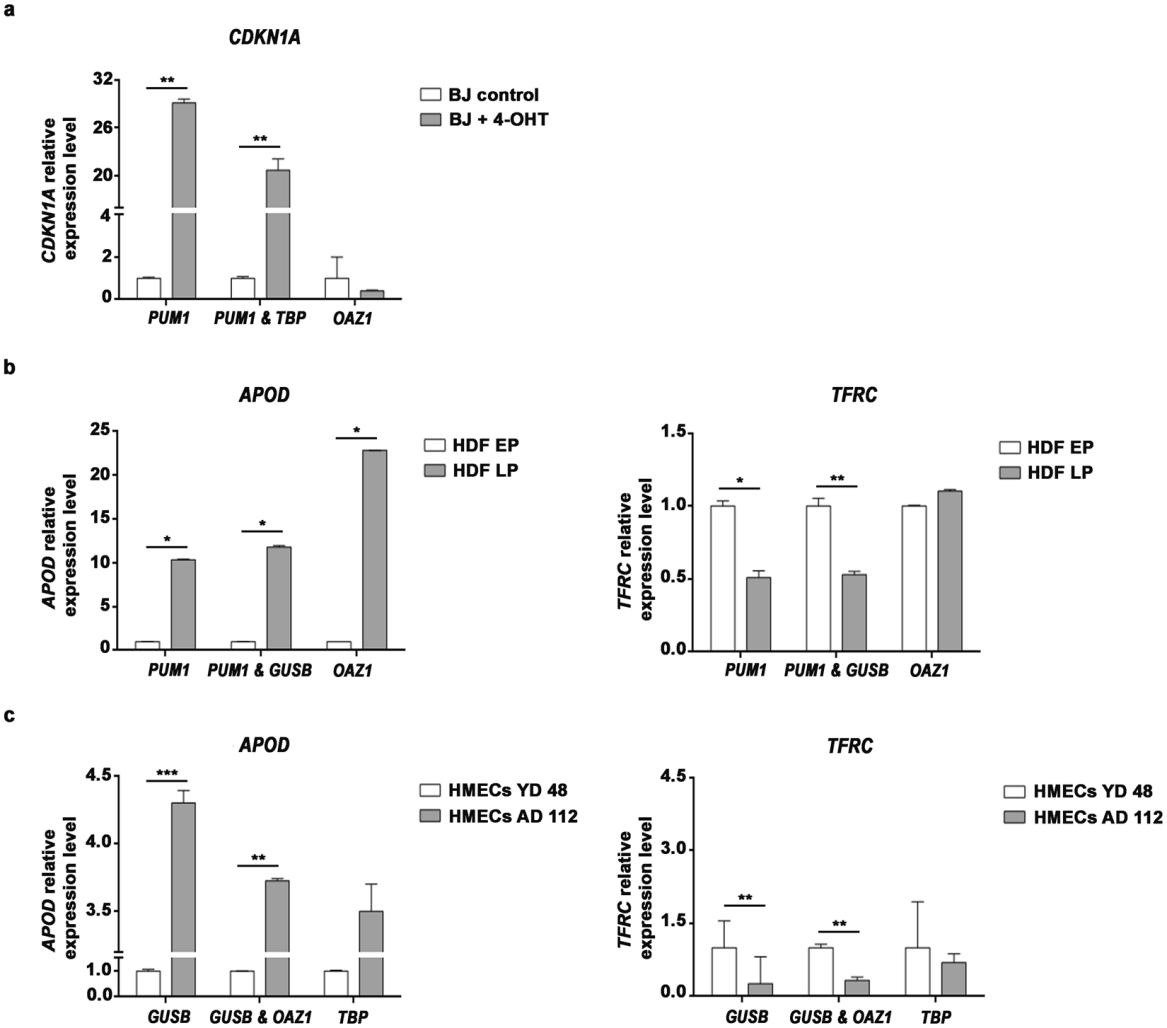
Expression levels of target genes normalised to different reference genes. Gene expression levels were normalised to the indicated reference gene or gene pair in the X axis. (**a**) Relative expression levels of *CDKN1A* in control and in senescent BJ fibroblasts. (**b**) Relative expression of *APOD* and *TFRC* in EP and LP HDFs and (**c**) in HMECs from a young donor and an aged donor (YD 48R & AD 112R, respectively). Relative fold changes in gene expression were obtained according to the 2^−ΔΔCt^ method. Abbreviations: 4-OHT: 4-Hydroxytamoxifen; HDF: Human Dermal Fibroblasts; EP: early passage (<10); LP: late passage (>20); HMECs: Human Mammary Epithelial Cells; YDs: young donors; ADs: aged donors; OIS: oncogene-induced senescence. Data are presented as mean and SD. *P*-values obtained from pairwise comparisons using Welch’s t test are shown (*p < 0.05, **p < 0.01, ***p < 0.001).

Among the various features of senescence, increased expression of *CDKN1A* has been widely used as a biomarker to identify senescent cells^44^. Therefore, we decided to check the *CDKN1A* expression profile in control and senescent BJ fibroblasts. Normalisation with the single most stable reference gene, *PUM1*, resulted in a significant increase of the *CDKN1A* expression level in senescent BJ fibroblasts compared to control BJ fibroblasts (Welch’s t-test; *p* = 0.0028) (Fig. 3a). The expression of *CDKN1A* was also significantly upregulated in senescent BJ fibroblasts after normalisation to the recommended reference gene pair, *PUM1* and *TBP* (Welch’s t-test; *p* = 0.0046) (Fig. 3a). These findings confirm that the combination of *PUM1* and *TBP* was also reliable for analysing expression changes of target genes during OIS (Fig. 3a). However, when the least stable gene, *OAZ1*, was used for data normalisation, no statistical differences in *CDKN1A* expression were detected between normal and senescent cells. In fact, the *CDKN1A* expression pattern exhibited an opposite trend, decreasing in senescence instead of increasing (Fig. 3a) when *OAZ1* was used as the reference gene.

A variety of genes have been shown to be differentially expressed during aging, making them potential markers of aging. Significant overexpression of *APOD* has been described during *in vitro*^45^ and *in vivo* aging^12^. Conversely, the transferrin receptor (*TFRC*) was shown to decrease with age^12^. Therefore, we analysed the expression profile of both of these age-related genes to confirm the reliability of the chosen reference genes in models of *in vitro* and *in vivo* aging.

During *in vitro* aging, independent of the reference genes used for normalisation, the expression levels of *APOD* were higher in LP than in EP HDFs, suggesting a very consistent age-dependent increase in the expression level of this gene (Fig. 3b). Regarding *TFRC* expression, normalisation with the most stable single gene, *PUM1*, revealed a decrease in *TFRC* expression in LP HDFs in comparison to EP HDFs (Welch’s t-test; *p* = 0.0261) (Fig. 3b). Furthermore, after normalisation with the most reliable gene pair, *PUM1* and *GUSB*, a decrease in *TFRC* expression in LP HDFs compared to EP HDFs was again observed (Welch’s t-test; *p* = 0.0098) (Fig. 3b). In contrast, using the least stable reference gene for normalisation, *OAZ1*, statistical differences in *TFRC* levels between young and old cells were not detected (Fig. 3b).

To validate reference genes for *in vivo* aging, we compared the expression profiles of *APOD* and *TFRC* between HMECs from one representative young and one representative aged donor. Using the suggested most stable single reference gene, *GUSB*, we observed that *APOD* was upregulated in HMECs from the aged donor compared to HMECs from the younger one (Welch’s t-test; *p* < 0.001). Moreover, *TFRC* was downregulated in HMECs from the aged donor (Welch’s t-test; *p* = 0.0018) (Fig. 3c). Similar results were obtained upon normalisation with the recommended reference gene pair, *GUSB* and *OAZ1* (Fig. 3c). Using both reference genes for normalisation, a higher expression of *APOD* was found in HMECs from the aged donor compared to HMECs from the younger one (Welch’s t-test; *p* = 0.0096). In the case of *TFRC*, its expression was again downregulated in HMECs from the aged donor (Welch’s t-test; *p* = 0.0091) (Fig. 3c). Finally, when using *TBP*, the least stable reference gene, both *APOD* and *TFRC* expression levels showed large variations, reflected by a high SD value, and no statistical differences were displayed between the age groups (Fig. 3c).

## Discussion

Gene expression analysis has become one of the most powerful tools in many fields of biological research, and RT-qPCR has emerged as the most frequently used approach for highly sensitive and precise quantification of gene expression. The accuracy of RT-qPCR results strongly depends on a careful selection of appropriate reference genes for normalisation of gene expression. Many studies have demonstrated that the expression of commonly-used reference genes varies among different cell types, tissues and experimental conditions^24,30,38^. Since there are no universal reference genes for accurate normalisation, it is crucial to specifically select the most suitable reference gene for each experimental design.

The aging process is accompanied by gene expression changes that lead to a decline of many cellular functions and to the development of age-related diseases^1^. Therefore, to obtain an unbiased comparison of gene expression profiles during aging, it is vital to select the appropriate reference genes. Also, expression of these reference genes may vary among the aging models used. In this study, we evaluated the stability of potential reference genes and selected the most suitable ones for gene expression analysis in three different aging models: OIS, *in vitro* and *in vivo* aging.

We identified *PUM1* and *TBP* as the most stable pair of reference genes for OIS models. Expression analysis of the senescent marker *CDKN1A*^44^, after normalisation to *PUM1* and *TBP*, revealed increased *CDKN1A* expression during OIS. Increased expression levels of *CDKN1A* has been previously described in IMR-90 *ER:RAS* fibroblasts, a classical model used to study OIS in culture, using *GAPDH* or *ACTB* as reference genes^46^. However, we show that these genes display intermediate or low stability values during OIS in BJ fibroblasts. In this regard, experiments of single-cell RT-qPCR have demonstrated changes in *GAPDH* expression in IMR-90 fibroblasts after bleomycin-induced senescence^47^. Additionally, *GAPDH* and *ACTB* have shown considerable variations in fibroblasts in which senescence was induced using different methods^29^. These facts emphasise the need to specifically select reference genes depending on the senescence model and on the cell type. Indeed, together with the instability of classical reference genes, the potential use of new reference genes in a strain‐dependent manner has been recently reported in senescence studies^29^. Hence, we strongly advise to choose appropriate genes for every senescent experiment, but we suggest *PUM1* and *TBP* as a starting point for future OIS studies in BJ human fibroblasts.

We found *GUSB* and *PUM1* to be the most stable reference genes for the *in vitro* aging model and *GUSB* and *OAZ1* were determined as the most suitable reference genes for the *in vivo* aging model. Supporting our results, studies using white blood cells from aged mice and human peripheral blood mononuclear cells from young and aged donors also identified *GUSB* as the most stable reference gene^28,48^. Considering *in vitro* aging, it should be noted that the two most stable reference genes during this process in HDFs coincided with the most stable reference genes for *in vivo* aging in HMECs (*GUSB*) and for senescent BJ fibroblasts (*PUM1*). These results suggest that *in vitro* aging is a model that might share expression characteristics with both the senescence and *in vivo* aging models. Indeed, several studies have used serially cultured somatic cells as an aging-like model that mimics some of the cellular and molecular alterations related to organismal aging^40,49^. Nevertheless, it should be considered that these reference genes have been identified using human fibroblasts and HMECs, and we cannot reject that using other cell types may cause changes in the appropriate candidate pair. In both *in vivo* and *in vitro* aging models, validation of the most stable reference gene was performed by analysing age-related expression changes of *APOD* and *TFRC*. In both *in vitro* and *in vivo* aging, *APOD* expression increased with age while *TFRC* expression was reduced, which has already been described in previous studies^12^, validating our reference genes. Finally, the MIQE guidelines suggest the use of at least two reference genes and to test whether more than two are necessary^42^. Considering our results, in all the three models tested in our study, at least two reference genes are enough for a strong and reliable normalisation in RT-qPCR experiments.

Remarkably, we observed that most of the candidate reference genes exhibited strong variations in *in vivo* aged cells. These results are in agreement with previously published studies that demonstrated an increased cell-to-cell transcriptional variability in lymphocytes^50^ and cardiomyocytes^51^ from aged mice. Age-related heterogeneity of gene expression has also been described in human aging after analysing microarray data sets, including data from the kidney, skeletal muscle and the cerebral cortex^52^. Accumulation of senescent cells in aged tissues has been associated with the loss of their regenerative capacity and the subsequent deterioration of their physiological functions^6,7^. In this study, we analysed gene expression levels of cells isolated from aged human epithelial mammary tissue. The fact that aged tissues comprise a mixed population of replicative senescent cells, young cells and *in vivo* aged cells could explain the age-associated heterogenous gene expression profiles described by others^47,51^. Therefore, we hypothesise that at least part of the large variations in gene expression detected in HMECs during *in vivo* aging are due to the inherent heterogeneity of the *in vivo* aged tissue composition.

Finally, it is worth mentioning that *GAPDH* is one of the most commonly used reference genes, and it has been identified as a reliable reference gene for comparisons between young and old donors of human skeletal muscle^27^. Conversely, *GAPDH* showed expression variability in our three aging models and occupied a middle to low position in the stability ranking of each aging model, again demonstrating the importance of reference gene selection in these types of studies. The intermediate stability value of *GAPDH* supports its extended use as a relatively safe reference gene choice, which generally ensures confidence in the results of RT-qPCR assays. However, variability of *GAPDH* expression in aging processes may impede the detection of subtle expression variations in target genes when using *GAPDH* as reference gene.

In summary, we demonstrate that expression of reference genes changes in a specific manner depending on the aging model, and thus it could be concluded that each aging process needs its own subset of reference genes for the reliable normalisation of RT-qPCR data. This study underlines the importance of selecting stable reference genes to correctly quantify gene expression levels during aging. Therefore, this report should be regarded as a guideline for future gene expression analysis, which ultimately leads to a better understanding of the basis of human aging.

## Materials and Methods

### Cell culture

HDFs and BJ fibroblasts were cultured in Dulbecco’s Modified Eagle’s Medium (Biowest, Riverside, MO, USA) and supplemented with 10% fetal bovine serum, 1% GlutaMAX and 1% penicillin-streptomycin (Thermo Fisher Scientific Inc., Waltham, MA, USA). Finite lifespan pre-stasis HMECs were a kind gift from Martha Stampfer from Lawrence Berkeley National Laboratory. HMECs were obtained from reduction mammoplasty tissue of 5 donors: 48R (16 years old), 240L (19 years old), 112R (61 years old), 122L (66 years old) and 429ER (72 years old); or peripheral non-tumour containing mastectomy tissue of 1 donor: 353P (72 years old)^53^. Donors were classified depending on age into two groups: young donors (YDs, ≤19 years old) and aged donors (ADs, ≥61 years old). HMECs were cultured using M87A medium supplemented with cholera toxin (Sigma‐Aldrich, St. Louis, MO, USA) and oxytocin (Bachem, Torrance, CA, USA), with the addition of 100 U/mL penicillin and 100 µg/mL of streptomycin (Thermo Fisher Scientific Inc., Waltham, MA, USA)^54^. All the experiments with HMEC cells were performed with population doublings < 20, calculated from passage 2. Incubation conditions for all cell types were 37 °C and 5% CO_2_ atmosphere. Immortalised BJ human fibroblasts expressing *ER-RASval12* (BJ hTERT ER:RAS) were kindly provided by Maite Huarte’s laboratory at CIMA, *Universidad de Navarra*, who generated the cells by infecting BJ fibroblasts (purchased from ATCC, Manassas, VA, USA) with lentiviral particles containing hTERT and ER:RAS. HDFs were commercially obtained from Cell Applications (San Diego, CA, USA).

### Senescence induction and detection

Exponentially growing BJ hTERT *ER:Ras* fibroblasts were treated with 200 nM of 4-OHT (Sigma‐Aldrich, St. Louis, MO, USA) for 8 days to induce senescence. After 4-OHT treatment, detection of SA-β-galactosidase activity was performed following the Debacq-Chainiaux *et al*. protocol^55^. Briefly, cells were fixed with 2% formaldehyde-0.2% glutaraldehyde in 1x PBS for 10 min at room temperature, washed with 1x PBS and treated for 12 h with H_2_O containing 40 mM citrate–phosphate buffer (pH 6), 2 mM MgCl_2_, 150 mM NaCl, 1 mg/ml of X-Gal, and 5 mM potassium ferricyanide and potassium ferrocyanide. This treatment results in the presence of a blue-dyed precipitate in senescent cells. Cells were subsequently washed with 1x PBS, methanol and distilled water. Pictures identifying senescent cells were captured with an IX71 optic inverted phase contrast microscope that was equipped with a DP20 camera and cell^A software (Olympus, Hamburg, Germany). A total of 100 cells were captured and quantified per condition.

### RNA extraction and cDNA synthesis

Confluent cells were washed once with ice cold PBS. Then, PBS was aspirated and TRIzol reagent (Thermo Fisher Scientific Inc., Waltham, MA, USA) was added. After thoroughly mixing, extracts were kept at −80 °C until total RNA from all samples was collected. After thawing TRIzol extracts, chloroform was added at 1:5 (v/v), and RNA was separated from DNA and proteins by centrifugation. 200 μL of the aqueous phase was then mixed with the same amount of lysis solution of the Maxwell RSC simply RNA kit in conjunction with the Maxwell system (Promega, Madison, WI, USA). The RNA concentration and purity were evaluated with a NanoDrop 2000 spectrometer (Thermo Fisher Scientific Inc., Waltham, MA, USA). RNA samples with an absorbance ratio OD 260/280 between 1.8–2.1 and OD 260/230 between 2–2.2 were used for further analysis. The quality of the RNA samples was checked by on-chip electrophoresis on the Agilent 2100 Bioanalyzer following the manufacturer’s protocol (Agilent Technologies, Santa Clara, CA, USA). The RNA integrity number (RIN) for all the RNA samples was 10, which is the maximum RNA integrity value.

Single-stranded cDNA was synthesised from 1 µg of total RNA in a final volume of 20 µL. For this purpose, the iScript cDNA synthesis kit (Bio-Rad, Hercules, CA, USA) was used and the manufacturer’s instructions were followed. cDNA was stored at −20 °C for future use. The cDNA products were tested for genomic DNA contamination using agarose gel electrophoresis.

### Selection of reference genes and primers design

Eight candidate reference genes were selected to test their stability among different aging processes. From the eight candidate reference genes, *ACTB*, *OAZ1* and *TMEM199* primer pairs were obtained from the literature^29,30^. The rest of primer sets were designed using Primer3 online software^56^, and after *in silico* validation with UCSC Genome Browser (https://genome.ucsc.edu), were purchased from Condalab (Metabion, Munich, Germany).

### Quantitative PCR and primer efficiency calculations

Quantitative PCR reactions were performed using the universal SYBR Green Supermix (Bio-Rad, Hercules, CA, USA) on a CFX96 thermal cycler with Bio-Rad CFX Manager software (Bio-Rad, Hercules, CA, USA). 50 ng of cDNA were used for each reaction. The amplification program was initiated at 95 °C for 3 min followed by 40 cycles of 10 s at 95 °C and 30 s at 60 °C. After amplification, an additional thermal denaturising cycle (temperature ranged between 65 °C and 95 °C in 0.5 °C increments) was performed to obtain the melting curves of the RT-qPCR products and verify amplification specificity. The reactions for each sample were run in triplicates, unless otherwise specified. RT-qPCR primer efficiency was tested for each primer pair using 10-fold serial dilutions of cDNA chosen among the samples. The mean threshold cycle (Ct) values for each serial dilution was plotted against the logarithm of the cDNA dilution factor. RT-qPCR primer efficiencies were calculated using the following equation:

$$E=(({10}^{\frac{-1}{slope}})-1)\ast 100$$

The efficiency of all designed primer pairs ranged from 80% to 110%, which is considered the optimal efficiency value^42,43^.

### Data analysis

To evaluate the stability of the candidate reference genes, two Add-in Microsoft Excel algorithms were applied, NormFinder^37^ and geNorm^38^. NormFinder provides a ranking of tested genes based on a stability value calculated from both intra- and intergroup variations of gene expression. GeNorm calculates the *M* value, a stability measure for each reference gene. A low *M* value reveals higher expression stability. Genes with an *M* value above 1.5 were considered inappropriate for normalisation. The algorithm identifies the two most stable reference genes by stepwise exclusion of the least stable gene. GeNorm also calculates the number of genes required for an optimal normalisation. Additionally, it compares the pairwise variation (V) of the two most stable genes with the remaining genes; thus, it calculates the V value of V_n_/V_n+1_ between two sequential normalisation factors. A variation of V_n_/V_n+1_ below 0.15 suggests that no additional reference gene is required for normalisation.

### Validation of candidate reference genes

To validate the reliability of the reference genes for RT-qPCR data normalisation, the relative expression of *CDKN1A*, *APOD* and *TFRC* was analysed. The primers’ specifications for these genes are listed in Supplementary Table 1. Expression of these genes was analysed using the most stable single and pair of reference genes and also the least stable gene. Normalisation of RT-qPCR data using two reference genes was performed with the geometric mean of the multiple reference genes (f) applying the formula below^38,57^ (*goi*, gene of interest; *ref*, reference gene):

$${\rm{Normalised}}\,{\rm{relative}}\,{\rm{quantities}}=\frac{{2}_{goi}^{\Delta Ct,goi}}{\sqrt[f]{{\prod }_{i=1}^{f}{2}_{re{f}_{i}}^{\Delta Ct,re{f}_{i}}}}$$

A 100% PCR efficiency was assumed (reflected by a value of 2 at the base of the exponential function). Relative fold changes in gene expression were obtained according to the 2^−ΔΔCt^ method^58^.

### Statistical analysis

Statistical procedures and graph plotting were conducted in GraphPad Prism 6.01 (GraphPad Software, La Jolla, CA, USA). The coefficient of variation (CV) is defined as the ratio of the standard deviation to the mean. The Chi-square test was used to compare differences between the frequency of senescent cells. Means of different groups were compared and analysed using one-way ANOVA, followed by Bonferroni’s post-hoc test to generate adjusted *p*-values or Welch’s t-test. Differences were considered statistically significant when the *p*-value was less than 0.05.

### Ethical statement

The authors declare that all methods were carried out in accordance with relevant guidelines and regulations and that all experimental protocols were approved by *Universitat Autònoma de Barcelona*. HMEC specimens were obtained in the laboratory of Martha Stampfer between 1977–1981. This was before the current Institutional Review Board (IRB) regulations were in place, and consent at that time was covered by the hospitals’ consent forms, which allowed the pathologists to use or distribute discarded surgical material at their discretion.

## Supplementary information

Supplementary Figures and Tables
