# Supplementary material for: Identification of reference genes for RT-qPCR data normalisation in aging studies
Source: Sci Rep. 2019 Sep 27;9:13970. doi: 10.1038/s41598-019-50035-0 (PMC6764958; doi:10.1038/s41598-019-50035-0)
Supplement: Supplementary file 1 — Supplementary Figures and Tables [file 41598_2019_50035_MOESM1_ESM.docx]

**Identification of reference genes for RT-qPCR data normalisation in aging studies**

**Lourdes González-Bermúdez^1,^** ^†^**, Teresa Anglada^1,^** ^†^**, Anna Genescà^1^, Marta Martín Flix^1,^** ^§,^* **and Mariona Terradas Ill^1,2,^** ^§,^*****

^1^Departament de Biologia Cel·lular, Fisiologia i Immunologia, Facultat de Biociències, Universitat Autònoma de Barcelona, Bellaterra, Spain

^2^Current address: Hereditary Cancer Program, Catalan Institute of Oncology, IDIBELL, Hospitalet de Llobregat, Barcelona, Spain

^†^These authors contributed equally to the study.

^§^These authors contributed equally to this work.

^*^Corresponding author: correspondence should be addressed to MTI (email: mterradas@idibell.cat) or MMF (email: Marta.Martin@uab.cat)

**Supplementary Figures**


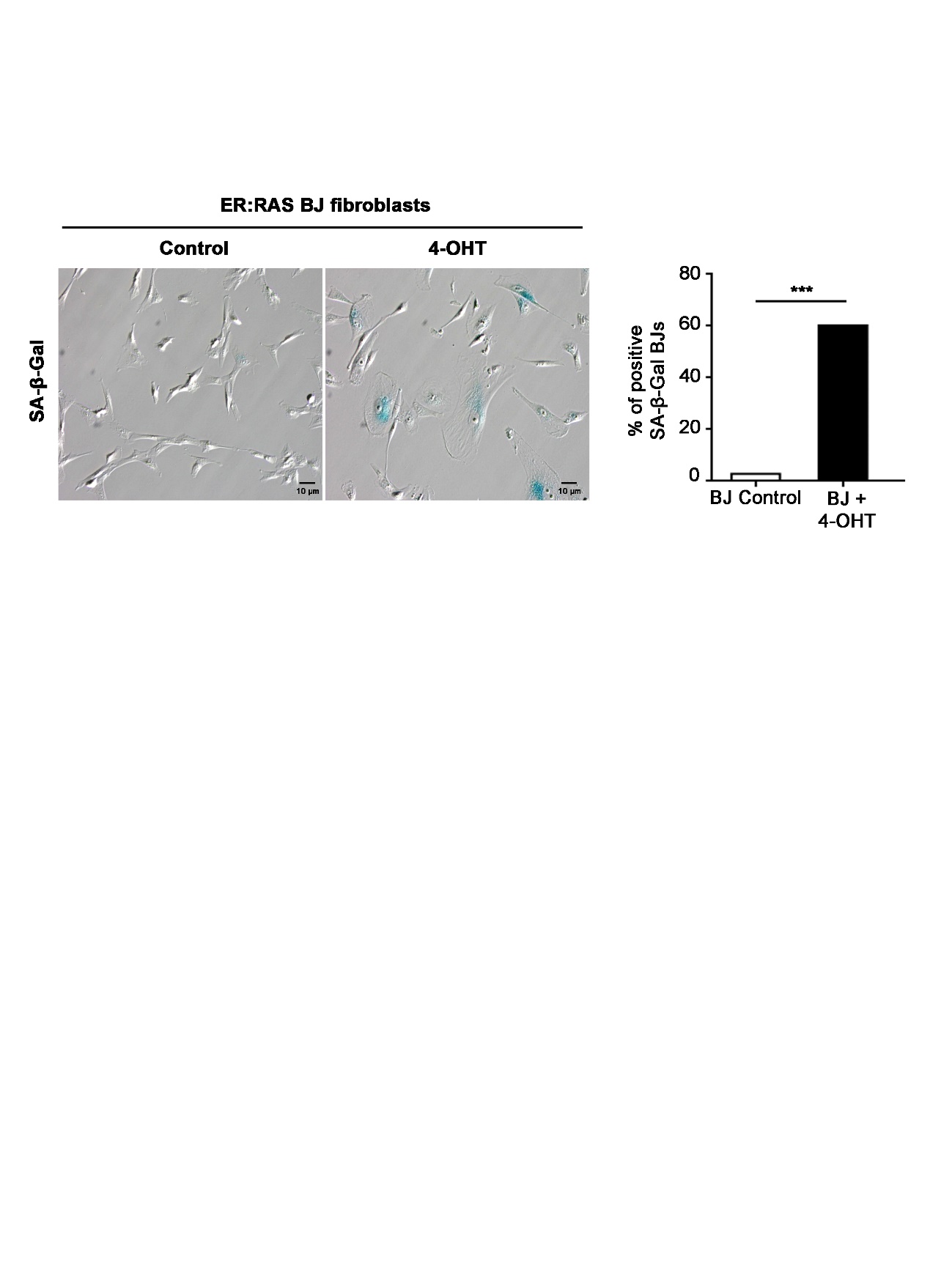


**Supplementary Figure 1. Oncogene-induced senescence in BJs fibroblasts.** Percentage of positive SA-β-Gal ER:RAS BJ fibroblasts in control conditions and after 4-Hydroxytamoxifen (4-OHT) treatment. The *p*-value indicates the Chi-square test significant level: ****p* < 0.001.


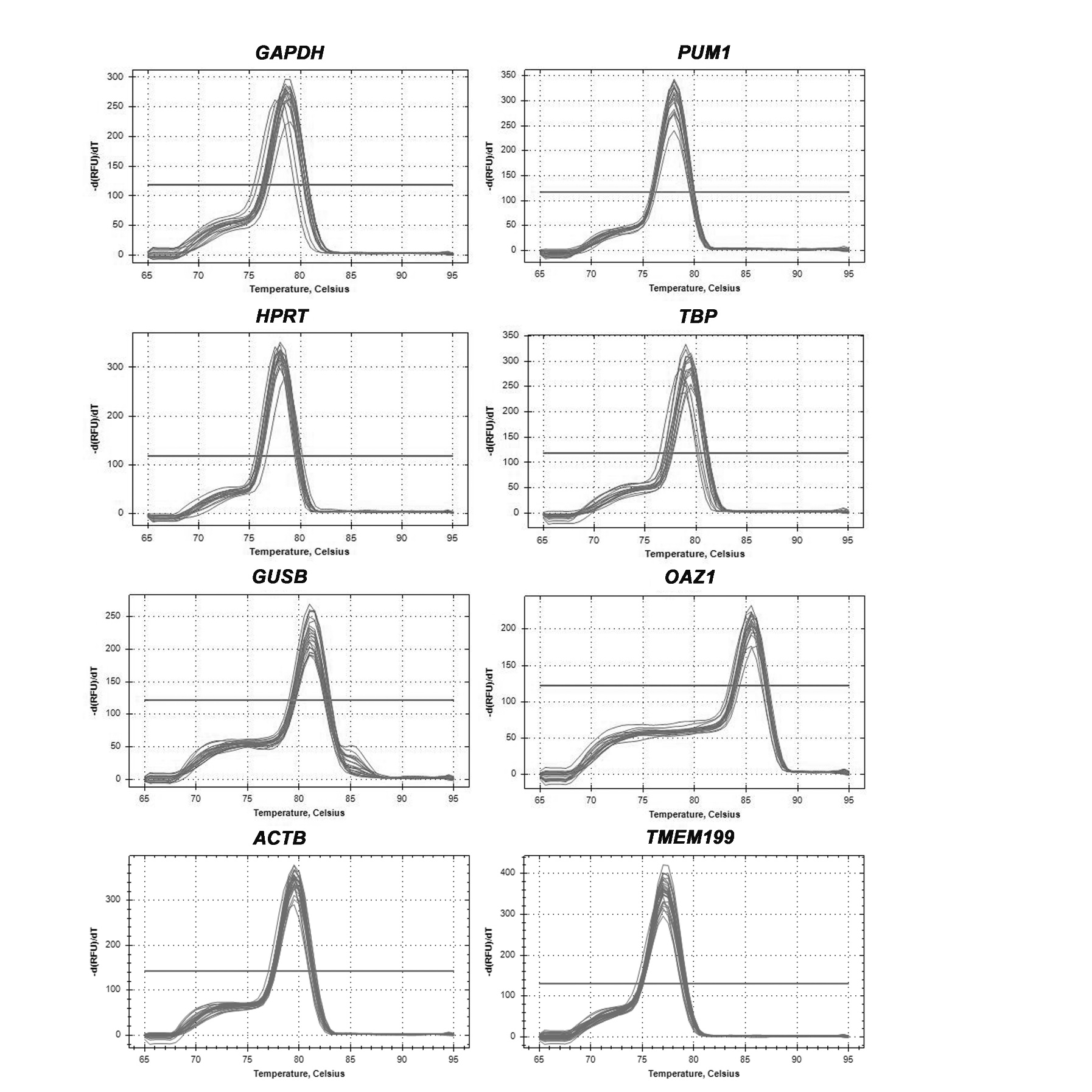


**Supplementary Figure 2. Melting curve analyses of all primers used in the study.** For each primer pair, only single peaks were observed, indicating the specificity of RT-qPCR amplification and the absence of primer dimers.

**Supplementary tables**

**Supplementary Table 1.** Primer details of validation genes.

| **Gene Symbol** | **ENSEMBL ID** | **Primer Sequence** | **Amplicon Size (bp)** | **Efficiency (%)** | **R^2^** |
| --- | --- | --- | --- | --- | --- |
| *CDKN1A* | ENSG00000124762 | F: CCGAAGTCAGTTCCTTGTGG  R: CATGGGTTCTGACGGACAT | 112 | 92.44 | 0.996 |
| *APOD* | ENSG00000189058 | F:GCAGCGTCCATTCTCAAAGG  R:GGCAGAGGGACAAGCATTTC | 133 | 109.83 | 0.998 |
| *TFRC* | ENSG00000072274 | F:GCAATCCTGATGACCGAG  R:TTTCCCATTGTTAACGCA | 127 | 94.49 | 0.999 |

Abbreviations: bp, base pair; F, forward; R, reverse; R^2^, correlation coefficient of the corresponding

standard curve.
